# Supplementary material for: Genome-Scale Metabolic Reconstruction, Non-Targeted LC-QTOF-MS Based Metabolomics Data, and Evaluation of Anticancer Activity of Cannabis sativa Leaf Extracts
Source: Metabolites. 2023 Jun 24;13(7):788. doi: 10.3390/metabo13070788 (PMC10385671; doi:10.3390/metabo13070788)
Supplement: Supplementary file 1 [file metabolites-13-00788-s001.zip › Supplementary Figures and Tables.pdf]

Supplementary Figures and Tables

Supplementary Figures

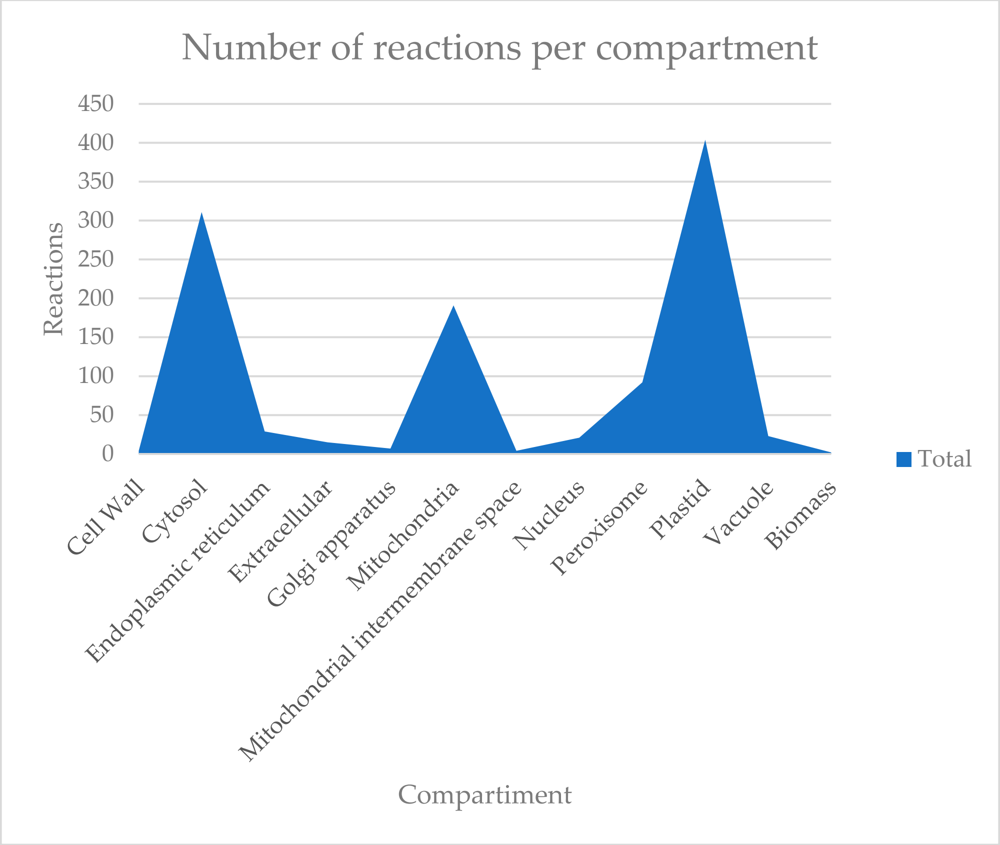

Supplementary Figure S1 Number of reactions per compartment in metabolic reconstruction.

Photosynthesis and Photorespiration:

Results of the flux predictions for these phenotypical states are related in Supplementary Fig 2. In the case of glycolysis, appropriate behavior is observed for some enzymes (*but not all*) that simulate a phenotypic state of the cell corresponding to photosynthesis and respiration.

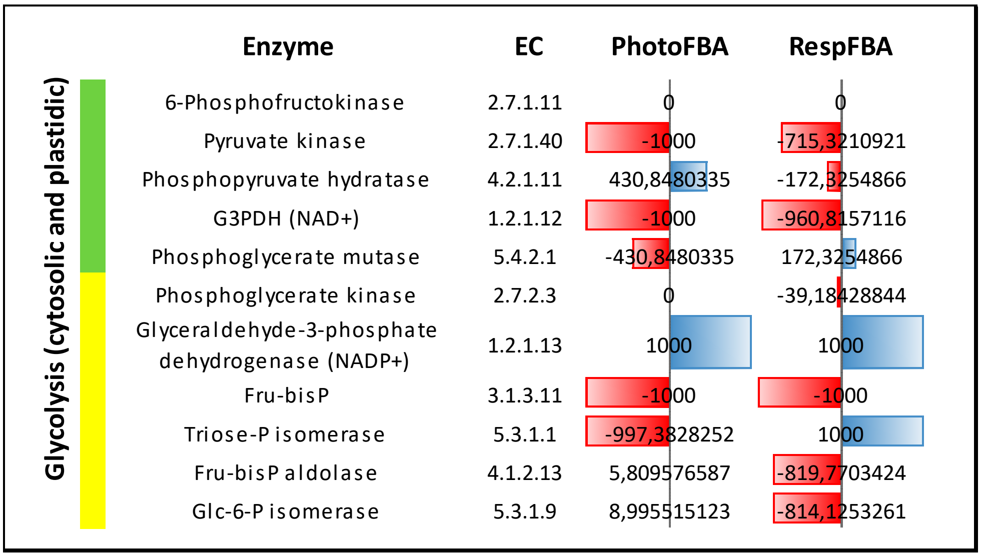

Enzyme	EC	PhotoFBA	RespFBA
6-Phosphofructokinase	2.7.1.11	0	0
Pyruvate kinase	2.7.1.40	-1000	-715,3210921
Phosphopyruvate hydratase	4.2.1.11	430,8480335	-172,3254866
G3PDH (NAD+)	1.2.1.12	-1000	-960,8157116
Phosphoglycerate mutase	5.4.2.1	-430,8480335	172,3254866
Phosphoglycerate kinase	2.7.2.3	0	-39,18428844
Glyceraldehyde-3-phosphate dehydrogenase (NADP+)	1.2.1.13	1000	1000
Fru-bisP	3.1.3.11	-1000	-1000
Triose-P isomerase	5.3.1.1	-997,3828252	1000
Fru-bisP aldolase	4.1.2.13	5,809576587	-819,7703424
Glc-6-P isomerase	5.3.1.9	8,995515123	-814,1253261

Supplementary Figure S2 Glycolysis in GEM reconstruction of *C. sativa*.

(A)

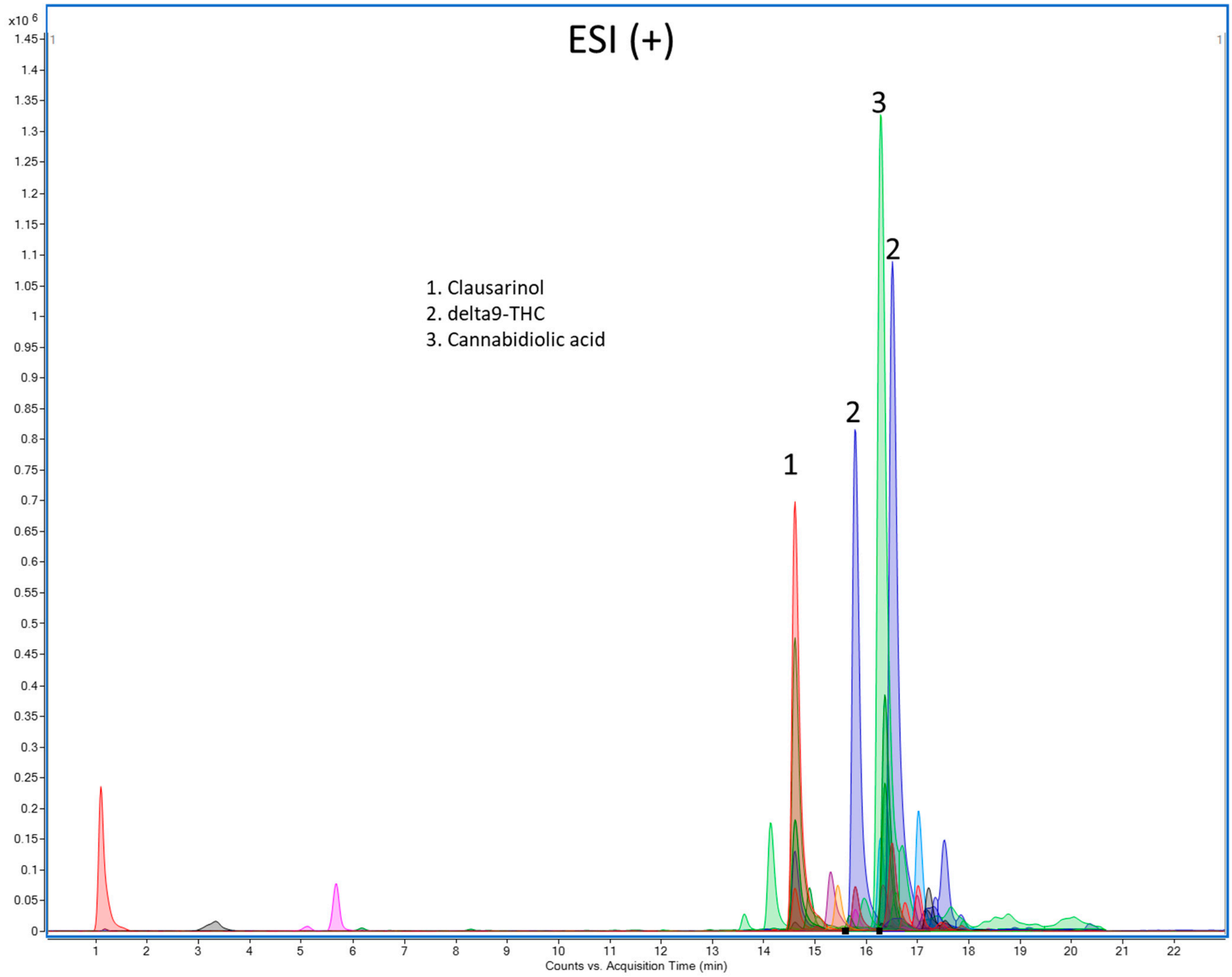

(B)

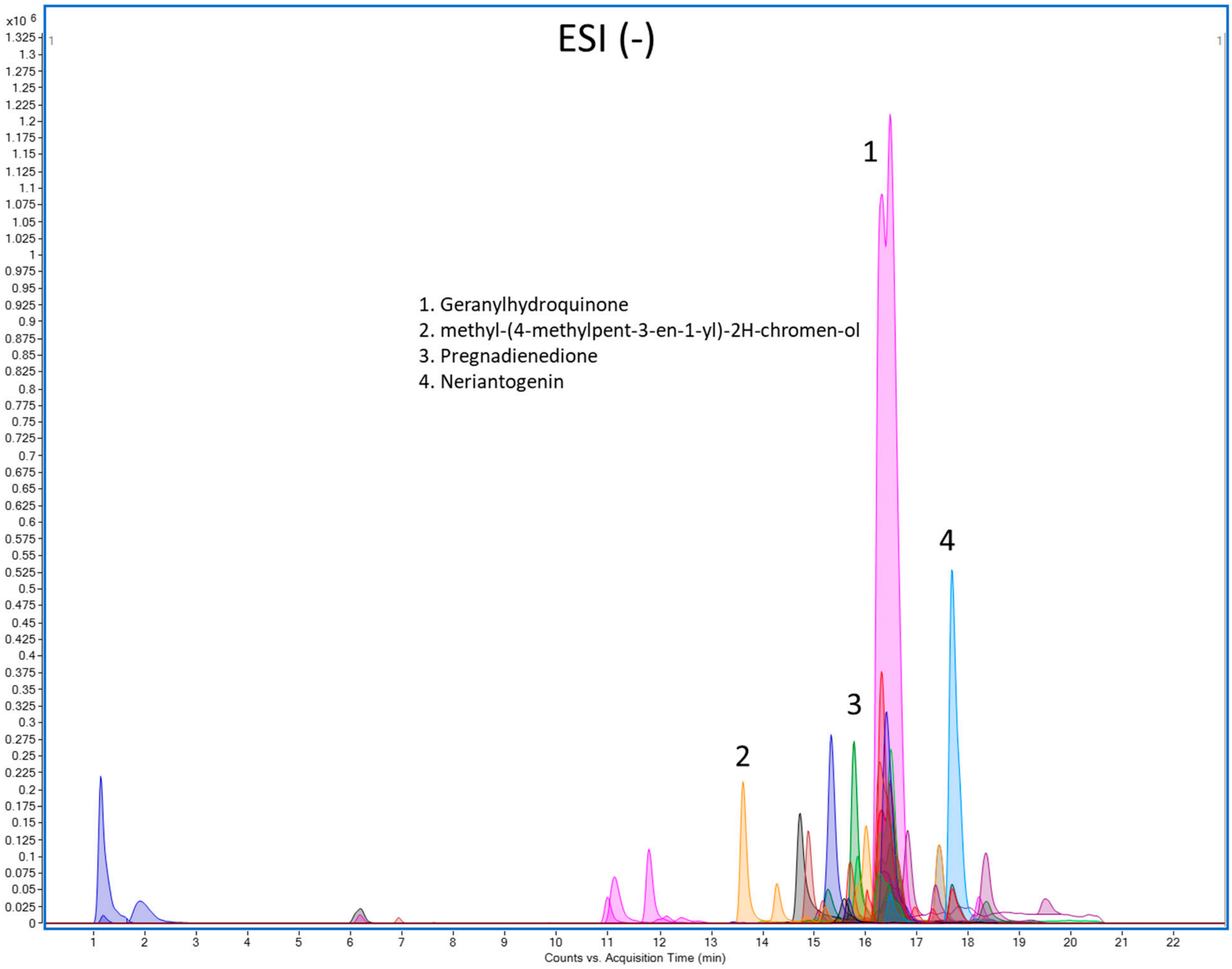

Supplementary Figure S3 Chromatogram of the main ions extracted from *C. sativa*. **(A)** ESI (+) detection mode. **(B)** ESI (-) detection mode.

.

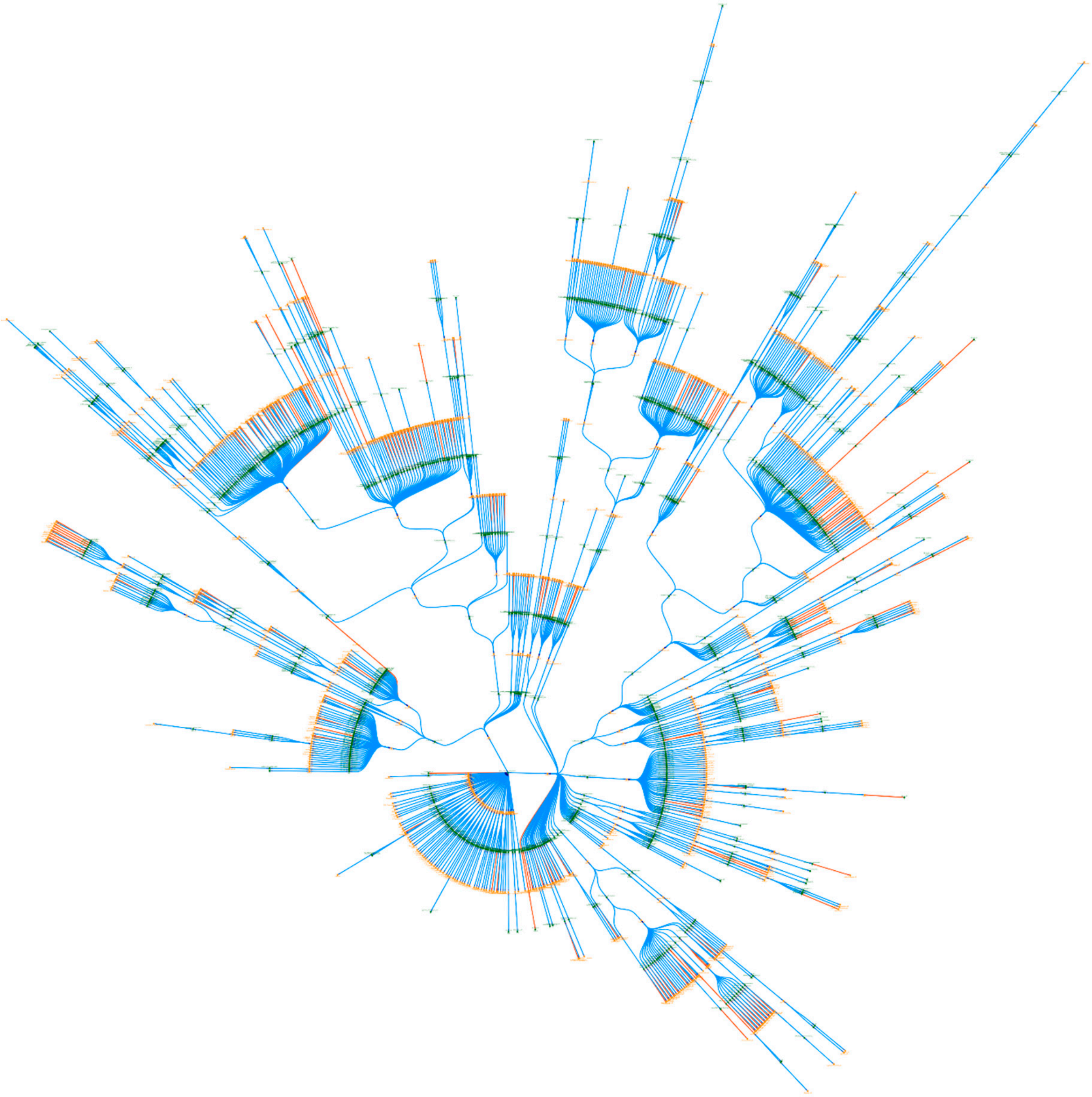

Supplementary Figure S4 Fluxer nodes and edge representation of metabolic reconstruction of *C. sativa* model.

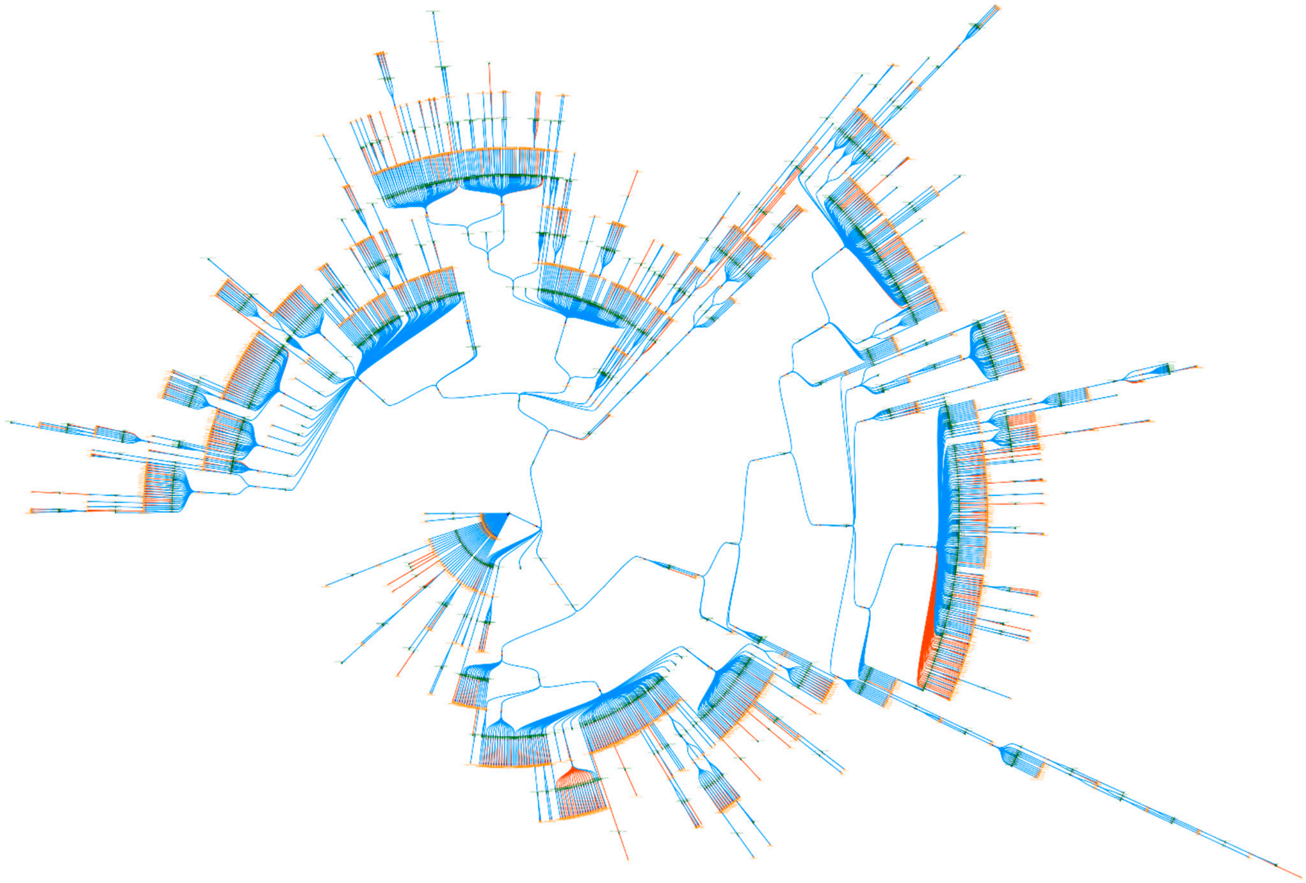

Supplementary Figure S5 Fluxer nodes and edge representation of metabolic reconstruction of Ar-aGEM model.

Supplementary Table

Supplementary Table S1. Biomass compounds in the objective function.

BiomassComponent	stoicBiomassFunction
'H2O_c0'	-30.0000
'Phosphate_c0'	30.0000
'PPi_c0'	0.2180
'H_plus__c0'	30.0000
'NAD_c0'	-0.0003
'ATP_c0'	-30.0270
'NADP_c0'	-0.0001
'ADP_c0'	30.0000
'NADH_c0'	-0.0002
'NADPH_c0'	-0.0001
'CoA_c0'	-0.0001
'UDP_c0'	0.7660
'UTP_c0'	-0.0270
'FAD_c0'	-0.0001
'FMN_c0'	-0.0001
'Pyridoxal_phosphate_c0'	-0.0001
'S_Adenosyl_L_methionine_c0'	-0.0005
'L_Methionine_c0'	-0.0490

'L_Malate_c0'	-0.0370
'L_Glutamate_c0'	-0.1300
'L_Glutamine_c0'	-0.0710
'L_Alanine_c0'	-0.1610
'GTP_c0'	-0.0260
'Oxaloacetate_c0'	-0.0757
'Citrate_c0'	-0.0130
'L_Aspartate_c0'	-0.0980
'CTP_c0'	-0.0260
'L_Asparagine_c0'	-0.0560
'L_Serine_c0'	-0.1000
'TPP_c0'	-0.0001
'Glycine_c0'	-0.1500
'L_Phenylalanine_c0'	-0.0740
'L_Lactate_c0'	-0.0390
'L_Tyrosine_c0'	-0.0490
'L_Threonine_c0'	-0.0960
'Glycerol_3_phosphate_c0'	-0.1300
'L_Cysteine_c0'	-0.0290
'Tetrahydrofolate_c0'	-0.0001
'10_Formyltetrahydrofolate_c0'	-0.0001
'5_10_Methylenetetrahydrofolate_c0'	-0.0001
'5_Methyltetrahydrofolate_c0'	-0.0001
'dATP_c0'	-0.0300
'5_10_Methenyltetrahydrofolate_c0'	-0.0001
'L_Proline_c0'	-0.0960
'cis_Aconitate_c0'	-0.0860
'UDP_xylose_c0'	-0.7660
'4_Coumarate_c0'	-0.5500
'dGTP_c0'	-0.0260
'TTP_c0'	-0.0300
'Ferulate_c0'	-0.0760
'5_Formyltetrahydrofolate_c0'	-0.0001
'dCTP_c0'	-0.0260
'Phosphopantetheine_c0'	-0.0010
'K_plus__c0'	-0.3070
'L_Tryptophan_c0'	-0.2400
'L_Valine_c0'	-0.1270
'L_Arginine_c0'	-0.1100
'Cl__c0'	-0.2100
'Palmitate_c0'	-0.3290
'L_Histidine_c0'	-0.3800
'L_Isoleucine_c0'	-0.0830
'L_Leucine_c0'	-0.1720
'L_Lysine_c0'	-0.0960
'ocdca_c0'	-0.0110

**'alpha_D_Glucose_c0'**	-1.5900
**'beta_D_Fructose_c0'**	-0.2271
**'beta_D_Ribofuranose_c0'**	-0.0377
**'BIOT_c0'**	-0.0001
**'Phytonadiol_d0'**	-0.0010
**'Oleate_c0'**	-0.0150
**'ubiquinol9_c0'**	-0.0001
**'Plastoquinol_9_c0'**	-0.0002
**'Biomass_c0'**	1.0000
